# Lactate clearance for death prediction in severe sepsis or septic shock patients during the first 24 hours in Intensive Care Unit: an observational study

**DOI:** 10.1186/2110-5820-3-3

**Published:** 2013-02-12

**Authors:** Philippe Marty, Antoine Roquilly, Fabrice Vallée, Aymeric Luzi, Fabrice Ferré, Olivier Fourcade, Karim Asehnoune, Vincent Minville

**Affiliations:** 1 Department of Anesthesiology and Intensive Care, CHU Toulouse, Université Toulouse III Paul Sabatier, Faculté de Médecine Toulouse-Rangueil, EA 4564-MATN, Institut Louis Bugnard (IFR 150), c, F-31000, France; 2 Department of Anesthesiology and Intensive Care, CHU Nantes, Nantes, France

**Keywords:** Sepsis, Lactate, Lactate clearance, Prognostic factor, Goal-directed therapy

## Abstract

**Background:**

This study was design to investigate the prognostic value for death at day-28 of lactate course and lactate clearance during the first 24 hours in Intensive Care Unit (ICU), after initial resuscitation.

**Methods:**

Prospective, observational study in one surgical ICU in a university hospital. Ninety-four patients hospitalized in the ICU for severe sepsis or septic shock were included. In this septic cohort, we measured blood lactate concentration at ICU admission (H0) and at H6, H12, and H24. Lactate clearance was calculated as followed: [(lactate_initial_ - lactate_delayed_)/ lactate_initial_] x 100%].

**Results:**

The mean time between severe sepsis diagnosis and H0 (ICU admission) was 8.0 ± 4.5 hours. Forty-two (45%) patients died at day 28. Lactate clearance was higher in survivors than in nonsurvivors patients for H0-H6 period (13 ± 38% and −13 ± 7% respectively, *p* = 0.021) and for the H0-H24 period (42 ± 33% and −17 ± 76% respectively, *p* < 0.001). The best predictor of death at day 28 was lactate clearance for the H0-H24 period (AUC = 0.791; 95% CI 0.6-0.85). Logistic regression found that H0-H24 lactate clearance was independently correlated to a survival status with a *p* = 0.047 [odds ratio = 0.35 (95% CI 0.01-0.76)].

**Conclusions:**

During the first 24 hr in the ICU, lactate clearance was the best parameter associated with 28-day mortality rate in septic patients. Protocol of lactate clearance-directed therapy should be considered in septic patients, even after the golden hours.

## Background

Severe sepsis and septic shock are a leading cause of death in the world [1]. An early strategy of quantitative resuscitation enables a decrease in mortality rate of septic patients, but few data are available for prolonged intensive care unit (ICU) strategy [2,3]. Despite the international recommendation of an early goal-directed therapy [4], based on optimization of mean arterial pressure, central venous pressure, urine output, and central venous oxygen saturation (ScvO_2_), mortality rate remains high in septic shock [5-7]. There is a need to test the prognostic value of factors that could be used for guiding therapy after the initial resuscitation.

Static blood lactate concentrations have already been tested in this purpose. In case of circulatory failure, such as severe sepsis, lactate concentration is a complex result of an anaerobic production, an aerobic production via the Na-K ATPase channel, and a decrease in lactate utilization [8-11]. However, several studies have described a correlation between baseline lactate concentration and mortality of ICU patients [12-15]. Although informative for severity assessment of septic patients, basal lactate rate is useless to guide therapy of such patients. Recently, lactate clearance in the first 6 hours was associated with an improvement of outcome in severe sepsis and septic shock patients and was proposed to guide treatment [16]. Lactate clearance as a goal of early sepsis therapy (in the first 6 hours), compared with central venous saturation, did not reduce the mortality of septic patients [17]. Longer duration of lactate clearance monitoring might result in better results, but there is a lack of data regarding lactate clearance in ICU after the first 6 hours of treatment for sepsis.

We hypothesized that lactate clearance at arrival in intensive care unit (after the golden hours in the emergency department) could remain predictive for outcomes. Thus, we investigated the prognostic value for death of lactate concentration, lactate clearance, and parameters derived from ScvO_2_ during the first 24 hours in ICU.

## Materials and methods

### Setting

According to French legislation (articles L.1121-1 paragraph 1 and R1121-2, Public Health Code), no informed consent is needed to use data for an observational study and informed consent from patients or relatives was waived by the institutional review board. No change in our current clinical practice (all measurements were routinely required in septic shock patients) and no randomization was performed.

### Design of the study

The study was a prospective, observational case series of adult patients. The study was conducted from April 2006 to may 2007 in a 16-bed University Hospital ICU. Consecutive patients admitted in this ICU for severe sepsis or septic shock, who were from the emergency department, were screened for the study. According to international recommendations [4], sepsis was defined as infection plus at least two criteria of systemic inflammation response. Severe sepsis was defined as sepsis plus sepsis-induced organ dysfunction or tissue hypoperfusion. Septic shock was defined as sepsis-induced hypotension (systolic blood pressure <90 mmHg or mean arterial pressure <70 mmHg) persisting despite adequate fluid resuscitation. A blood lactate concentration above 4 mmol.l^-1^ or oliguria (<0.5 ml.hr^-1^) defined hypoperfusion. Exclusion criteria were age ≤18 years, pregnancy, and ICU-acquired severe sepsis.

Patient management in the emergency department was performed as recommended in the international guidelines for septic shock [4]. The inclusion of the patient in our study began at the arrival in the ICU (H0), i.e., after the initial management in the emergency department. Patient management in intensive care unit was gone on to reach the international guidelines goals during the first 24 hr hospitalization [4]: 1) mean arterial pressure (MAP) ≥65 mmHg and diastolic arterial pressure (DAP) ≥40 mmHg; 2) ScvO_2_ ≥ 70%; (3) urine output ≥0.5 ml/kg/hr.

Our standard septic shock resuscitation treatment was administrated in sequence until the following goals were reached:

1. Diagnosis of septic shock with lactate measurement followed by 30–40 ml/kg crystalloid fluid loading over 1 hour.

2. Norepinephrine titrated to obtain a MAP ≥65 mmHg and a DAP ≥40 mmHg.

3. ScvO_2_ measurement: fluid loading of 500 ml crystalloid and/or colloid every 10 min until ScvO_2_ reached a value ≥70%.

4. Blood transfusion if ScvO_2_ remained ≤ 70% and hematocrit (Ht) ≤30%.

5. Dobutamine at a dose of 5 μg/kg/min if Ht ≥30% and ScvO_2_ ≤70%.

6. After 6 hr when lactate level remained higher than 2 mmol/l: cardiac output was measured by a Pulse Indexed Continuous Cardiac Output (PiCCo) monitor (Pulsion, Medical Systems AG, Munich, Germany) to optimize oxygen delivery (DO_2_) and reach an ScvO_2_ ≥70%: successive colloid “fluid challenge” of 500 ml over 15 min until CI variation during the challenge became less than 15%, transfusion if Ht ≤30% and ScvO_2_ ≤70%, dobutamine if ScvO_2_ remained lower than 70%.

7. If ScvO_2_ remained lower than 70% after this DO2 optimization, attempts to lower oxygen consumption were instituted (fever control and/or mechanical ventilation after tracheal intubation and/or increase in sedation and/or pain medication).

Others procedures within the first 24 hr:

1. After bacterial samples, eradication of any septic focus with early empiric broad-spectrum antibiotics and surgery when needed.

2. Prescription of low dose hydrocortisone if patients were treated by norepinephrine for more than 6 hr as recommended [4].

### Data collection

Diagnosis, age, gender, and calculated Simplified Apache Physiology Score (SAPS 2) were prospectively recorded. Blood lactate concentration, mean arterial pressure, cardiac index assessed with PiCCO®, arterial oxygen saturation (SaO_2_) measured on arterial blood gas, ScvO_2_ measured on a central venous blood sample (superior vena cava) were collected when patients arrived in ICU (H0) and successively at 6, 12, and 24 hours after admission (H6, H12, and H24 after the ICU admission). Survival was followed-up during 28 days. As previously described [16,17], the lactate clearance was defined by the equation [(lactate_initial_ - lactate_delayed_)/ lactate_initial_] × 100%. Lactate_delayed_ is blood lactate concentration measured at H6, H12, or H24. A positive value indicates a decrease in lactate rate. ScvO_2_ variation was defined by the equation [(ScvO_2delayed_ – ScvO_2initial_)/ScvO_2initial_] × 100%. Time of inclusion (H0) and study enrolment were considered as the time of ICU admission. The transit time in the emergency room between diagnosis of septic shock and enrollment in ICU was registered.

### Statistical analysis

Quantitative variables are presented as mean ± SD and qualitative data are given as number and percentage. Normal distribution of data was tested via Kolmogorov-Smirnov test. The values results were not normally distributed and thus were analyzed non-parametrically.

Survivors and nonsurvivors were compared by the Mann-Whitney *U* test for continuous variables and by Fisher’s exact test for categorical variables. To assess whether the lactates changed over time, Friedman’s test was used. When Friedman’s test was significant (*p* < 0.05), pairwise comparisons were made using Wilcoxon’s signed-rank test. All tests were two-sided, and *p* < 0.05 indicated a statistical significance. Because lactate and SvcO_2_ are numerical data, receiver-operating characteristics (ROC) curves and area under the curve (AUC) were computed. For mortality prediction, cutoff values for lactates, lactate clearance, and SvcO_2_ were chosen to correspond to the best respective Youden’s index calculated as follows: Youden’s index = sensitivity + specificity −1. Results are expressed for area under the ROC curves (AUC) as mean (95% confidence interval).

A logistic regression was performed to discriminate if SAPS II, lactate at H0, H6, H12, and H24, lactate clearance H0-H6, H0-H12, and H0-H24, and norepinephrine were independently correlated to the survival status. Goodness of fit of the model was assessed using the Hosmer-Lemeshow test. Statistical analysis was realized by SAS 9.1.3 software.

## Results

### Patient characteristics

From April 2006 to May 2007, 96 septic shock patients who were admitted in the ICU and who had been previously resuscitated in the emergency unit were considered eligible. Two patients were excluded (one for pregnancy and one for age <18 years). The mean time between severe sepsis diagnose and H0 (ICU admission) was 8.0 ± 4.5 hours.

We analyzed the data of 94 patients with severe sepsis or septic shock. Demographic data are provided in Table 1. All of the patients received sedation (Ramsay score between 3 and 5) and were mechanically ventilated. Norepinephrine was used in all patients. Thirty-one patients were treated with dobutamine. Forty-two (45%) patients died in the first 28 days. The mean SAPS 2 was 53 ± 16 in survivors versus 69 ± 15 in nonsurvivor patients (*p* < 0.01). No statistical difference was noticed for MAP and CO between survivors and nonsurvivors at any time.

**Table 1:** Demographic characteristics

	**Overall population (N = 94)**	**Survivors (N = 52)**	**Nonsurvivors (N = 42)**
Gender			
M/F (%)	56 / 44	59 / 41	54 / 46
Age (yr) (mean ± SD)	58 ± 16	55 ± 17	61 ± 15
SAPS 2 (mean ± SD)	60 ± 17	53 ± 16	69 ± 15^*^
Sepsis origin, N (%)			
Pulmonary	27 (29)	14 (27)	13 (31)
Digestive	26 (28)	18 (35)	8 (19)
Urinary	4 (4)	2 (4)	2 (5)
Other	37 (39)	18 (35)	19 (45)
Hemodynamic status on ICU admission (mean ± SD)			
Mean arterial pressure (mmHg)	66.5 ± 10.3	68 ± 11	64 ± 10
Cardiac index (L/min.m^2^)	3.4 ± 1	3.4 ± 0.9	3.4 ± 1.3
Norepinephrine (μg/kg/min)	0.62 ± 0.37	0.44 ± 0.34	0.77 ± 0.5^*^
Scv0_2_ (%)	73.3 ± 9.4	74.1 ± 9.2	72.3 ± 9.6

*ICU* intensive care unit; *SAPS 2* Simplified Acute Physiology Score; *ScvO*_*2*_ central venous oxygen saturation.

*p < 0.05 vs. survivors.

### Static blood lactate and lactate clearance in survivors and non survivors

**Figure 1:**
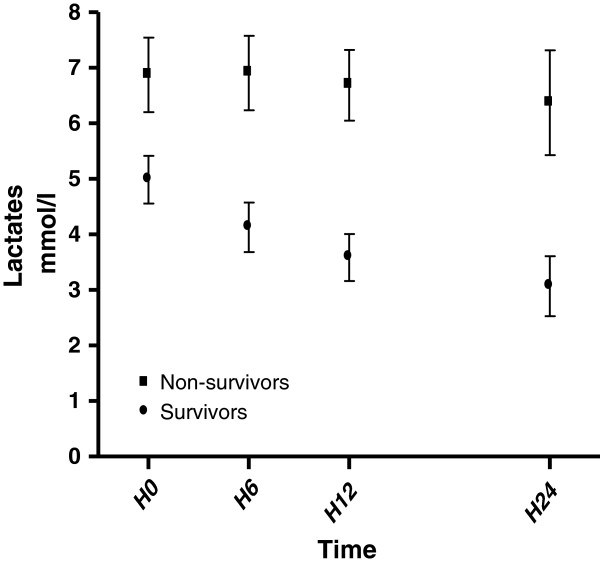
Time course of lactates in ICU.

The static blood lactate and lactate clearance are illustrated in Figure 1. There is a significant difference between H0 lactate value and H6, H12, or H24 lactate value in survivor group (*p* < 0.05 for each studied period). No significant difference was found in nonsurvivor group between H6, H12, and H24 lactate value compared with H0. Mean blood lactate concentrations were lower in survivors than in nonsurvivors patients at H0 (5 ± 3.1 mmol/L vs. 6.9 ± 4.3 mmol; *p* = 0.049). Afterward, blood lactate concentrations were lowers in survivors than in nonsurvivors at each studied time (4.1 ± 3.2 vs. 6.9 ± 4.3 at H6; 3.6 ± 2.9 vs. 6.7 ± 4 at H12 and 3 ± 3 vs. 6.4 ± 4.5 at H24. *p* < 0.05 for each studied period). Lactate clearance was 13 ± 38% in survivors and −13 ± 67% in nonsurvivors patients for the H0-H6 period (*p* = 0.021) and remained higher in survivors than in nonsurvivors for each studied period (42 ± 33% vs. −17 ± 76%; *p* < 0.001 for the H0-H24 period).

### Static SvcO_2_ and SvcO_2_ variation in survivors and nonsurvivors

The SvcO_2_ course is illustrated in Table 2. SvcO_2_ was not statistically different in survivors than in nonsurvivors patients at H0 and at each studied time. No significant SvcO_2_ variation was noticed for any period.

**Table 2:** Blood lactate concentrations and lactate clearances in survivors and non survivors during the first 24 hours in ICU

	**Survivors**	**Nonsurvivors**	***p***
**ScvO**_**2 **_**H0 (%)**	74.1 ± 9.2	72.3 ± 11.6	0.39
**ScvO**_**2 **_**H6 (%)**	76.2 ± 7.1	75.2 ± 10.9	0.57
**ScvO**_**2 **_**H12 (%)**	76.4 ± 7.2	73 ± 11.5	0.07
**ScvO**_**2 **_**H24 (%)**	76.1 ± 7.5	76 ± 10.8	0.9
**Variation ScvO**_**2 **_**H0-H6 (%)**	4.19 ± 9.4	4.4 ± 14.2	0.93
**Variation ScvO**_**2 **_**H0-H12 (%)**	2.9 ± 11.6	3.4 ± 10.8	0.83
**Variation ScvO**_**2 **_**H0-H24 (%)**	3.6 ± 14.9	6.5 ± 15.8	0.5

ScvO_2_ variation was defined by the equation [(ScvO_2delayed_ – ScvO_2initial_)/ScvO_2initial_] x 100%. Results expressed as mean ± SD. Pairwise comparisons were made using Mann-Whitney *U* test.

### Diagnostic Value of static blood lactate levels, blood lactate clearance and central venous saturation

For each period, ROC curves, AUC for lactate, and SvcO_2_ were calculated (Table 3). For each period, the most accurate cutoff value to distinguish survivors and nonsurvivors was evaluated (Table 3). Best ROC curves AUC were obtained for H24 static blood lactate (0.773; 95% CI 0.64-0.87) and H0-H24 lactate clearance (0.791; 95% CI 0.6-0.85). Scv0_2_-related ROC curves AUC revealed a very poor ability to predict day-28 mortality at any time.

**Table 3:** Area under the ROC curves for static lactate rates and lactate clearances

	**AUC**	**IC 95%**	**Threshold**	**Se.%**	**CI 95%**	**Sp.%**	**CI 95%**
**ScvO**_**2 **_**H0**	0.55	0.44–0.66	74%	53.19	38.1–67.9	64.86	47.5–79.8
**ScvO**_**2 **_**H6**	0.498	0.39–0.61	79%	72.34	57.4–84.4	42.11	26.3–59.2
**ScvO**_**2 **_**H12**	0.609	0.5–0.71	71%	88.89	75.9–96.3	36.84	21.8-54
**ScvO**_**2 **_**H24**	0.501	0.36–0.64	67%	90.32	74.2–97.8	29.17	12.7–51.1
**Variation ScvO**_**2 **_**H0-H6**	0.533	0.42–0.65	2.5%	54.55	38.9-69.6	58.82	40.7–75.3
**Variation ScvO**_**2 **_**H0-H12**	0.501	0.38–0.62	−5.2%	28.57	15.7–44.6	85.29	68.9-95
**Variation ScvO**_**2 **_**H0-H24**	0.598	0.45–0.73	4.2%	65.52	45.7-82	54.55	32.2–75.6
**Lactates H0**	0.613	0.5–0.71	5.4 mmol/l	76.92	63.2–87.5	54.76	38.7–70.1
**Lactates H6**	0.706	0.6–0.8	4.05 mmol/l	63.46	49–76.4	75.61	59.7–87.6
**Lactates H12**	0.729	0.62–0.82	4.2 mmol/l	75	60.4-86,3	66.67	49.8–80.9
**Lactates H24**	0.773	0.64–0.87	2.6 mmol/l	67.74	48.6–83.3	82.61	61.2–94.9
**Lac Clear H0-H6**	0.619	0.5–0.72	−7.7%	63.46	49–76.4	56.1	39.8–71.5
**Lac Clear H0-H12**	0.662	0.55–0.76	−44.6%	45.83	31.4–60.8	82.05	66.5–92.4
**Lac Clear H0-H24**	0.791	0.6–0.85	−2.1%	96.77	83.2–99.5	52.17	30.6–73.2

*AUC* area under the curve; *CI* confidence interval; *Se* sensibility; *Sp* specificity; *Lac Clear* lactate clearance.

### Multivariate analysis between the deceased and the survivors

Logistic regression found that only the lactate clearance H0-H24 and norepinephrine were independently correlated to a survival status with a *p* = 0.047 [odds ratio = 0.35 (95% CI 0.01-0.76)] and *p* = 0.038 [odds ratio = 2.59 (95% CI 1.05-6.41)] respectively (Table 4).

**Table 4:** Multivariate analysis between the deceased and the survivors

**Data**	***p***	**Odd ratio**	**95% CI**	
			**Inferior**	**Superior**
Lactates H0	0.4	1.57	0.55	4.45
Lactates H6	0.48	0.53	0.09	3.08
Lactates H12	0.17	5.3	0.48	59
Lactates H24	0.14	0.23	0.03	1.61
Lactates Cl H0-H6	0.61	2.19	0.27	83.06
Lactates Cl H0-H12	0.1	9.89	0.05	90.15
Lactates Cl H0-H24	0.047	0.35	0.01	0.76
SAPS 2	0.47	1.03	0.96	1.1
Norepinephrine	0.038	2.59	1.05	6.41

Goodness of fit was 0.73 with the Hosmer-Lemeshow test.

## Discussion and conclusions

The current results, obtained in a population of severe sepsis or septic shock patients, indicate and the prognostic value of lactate clearance in the first 24 hr in ICU. After initial resuscitation, ScvO_2_ may have poor ability to predict death at day 28.

Mixed central venous oxygen saturation is correlated to the central venous saturation and has been independently associated with mortality in septic shock, with threshold values supporting those published in guidelines [18]. In Varpula’s study a SvcO_2_ < 70% is independently associated with mortality. Moreover, even if it remains controversial, early goal-directed therapy (H0-H6) adapted to a target of ScvO_2_ has decreased the mortality rate in septic shock patients [19].

In the current study, neither static ScvO_2_ nor ScvO_2_ variation were predictive for death at day 28 at any time. Other studies performed in intensive care unit after initial resuscitation support these data [20,21].

These different results are not contradictory. ScvO_2_ appears to be a useful tool for initial resuscitation but is unable to distinguish survivors and nonsurvivors after this stage. Moreover, if SvcO_2_ <70% [18,19] is associated with mortality, it does not mean that SvcO_2_ ≥70% is associated with survival [22]. ScvO_2_ seems to be a necessary but not sufficient parameter to guide therapeutic intervention in ICU after initial resuscitation. In ICU-resuscitated patients, ScvO_2_ or mixed venous oxygen saturation is often larger than 70% despite evidence of abnormal tissue oxygenation. This oxygen extraction defects might be related to severe microcirculatory disorders [23] and/or mitochondrial damage and/or impairment of cellular respiration [24] resulting in most of the cases in elevated ScvO_2_ or SvO2 values [21,25]. Low levels reflect 1) an inadequate cardiac output with an excessive extraction of oxygen, 2) a low hemoglobin concentration, and/or 3) a low level of arterial oxygen pressure (PaO_2_). In contrast, high levels of ScvO_2_ means either 1) a very high oxygen delivery in excess of tissue requirements, and/or 2) decreased cellular consumption of oxygen (mitochondrial dysfunction), and/or 3) more rarely, a large arteriovenous shunt [26]. After early resuscitation, ScvO_2_ therefore may not be sufficient to guide titration of fluid loading and vasopressor therapy.

In a general ICU population, basal lactate concentration predicted the risk of death with a good accuracy [27]. In 2005, Varpula et al. analyzed hemodynamic parameters in septic shock patients and initial lactate concentration was higher in nonsurvivors (3.4 vs. 2.1 mmol/l respectively, *p* < 0.005) [18]. In a heterogeneous population of septic patients, blood lactate values, with a threshold of 4 mmo/l, were associated with in-hospital mortality but the predictive value was poor (reported AUC under the ROC curves = 0.56). The lactate concentrations were lower in survivors than in nonsurvivors all along the first 24 hr in ICU and lactate level at H0, even with a threshold of 6.2 mmo/l, indicating therefore a poor ability for death prediction in septic patients.

Several authors have reported that septic patients with the lowest lactate value at H24, even with the same initial lactate concentration, had the highest survival rate [28-30]. For Bakker et al., the “Lactime,” defined as the time passed with a lactate rate above a normal value, was more predictive for death than the initial lactate value [31]. In a cardiac arrest-resuscitated population, lactate levels at admission were not altered in survivors and nonsurvivors patients, whereas lactate clearances were superior in survivors [32]. In hemodynamically stable surgical patients, the association of an occult hypoperfusion with a prolonged hyperlactatemia has been associated with an increased mortality rate [14]. Dynamic assessment of metabolic values may be more efficient for death prediction than static values. Several studies, in severe septic patients, pointed out the value of blood lactate clearance in the first 6 hours of resuscitation for the prediction of day-28 survival [16,33], but no data are available for longer duration. In the current study, delta lactates until the 24^th^ hour in ICU were predictive for death. Finally, the calculation and interpretation of lactate clearance appeared useful even after the “golden hours” [3,4] and enable detection of patients with a high risk of death.

For septic patients, a lactate clearance-directed therapy in the first 6 hours appeared as efficient as ScvO_2_[17]. In a general ICU population, an 8-hour therapy adaptation to lactate clearance reduced the mortality rate in patients with hyperlactatemia compared with standard therapy [34]. No data are available for lactate-directed therapy for longer duration, and no target can thus be proposed. In the study of Jansen et al., a resuscitation adapted to a target of 20% decrease/2 hours for 8 hours decreased the mortality rate [34]. In another recent study, the addition of lactate clearance to the SSC resuscitation bundle is associated with improved outcome [35]. According to this study, absence of lactate clearance during the first 24 hours is associated with mortality in septic ICU patients and should lead to therapy intensification, even in patients who reach standard hemodynamic target.

Several limitations should be considered to interpret this study. First, it was an observational analysis whose results support an association and not necessarily causation. Second, data come from a single center and institution-specific variables may have influenced the present results. Moreover, in the current cohort, following international recommendations [4], patients with a low ScvO_2_ have received either dobutamine or transfusion as adapted and these therapeutic-adaptations may have limited our ability to confirm the value of ScvO_2_ as a prognostic factor.

In conclusion, during severe sepsis or septic shock, blood lactate concentration and lactate clearance are both predictive for 28-day mortality. Assessment of lactate clearance in the first 24 hours would be useful for tracking patients who remained at high risk of death despite achievement of recommended early goals determined by international recommendations. Protocols of prolonged lactate clearance-directed therapy should be evaluated in septic patients. 

## Competing interests

No financial support was required to perform the study. Authors declare no conflict of interest. This study was performed in the Department of Anesthesiology and Intensive Care, CHU Toulouse, Université Toulouse III Paul Sabatier, Faculté de Médecine Toulouse-Purpan, EA 4564-MATN, Institut Louis Bugnard (IFR 150), Toulouse F-31000, France.

## Authors’ contributions

PM and FV recorded all data and wrote the manuscript. AL and FF participated in the design of the study and performed the statistical analysis. AR, KA, OF and VM participated in its design and coordination and helped to draft the manuscript. All authors read and approved the final manuscript.
